# RNA Sequencing Technologies in Acute Lymphoblastic Leukemia: A Comparative Technical Review

**DOI:** 10.3390/cimb48080768

**Published:** 2026-07-28

**Authors:** Maria Koutra, Paschalis Evangelidis, Apostolia Papalexandri, Tasoula Touloumenidou, Emmanouel Hatzipantelis, Ioanna Sakellari, Eleni Gavriilaki, Athanasios Tragiannidis

**Affiliations:** 1 Hematology & BMT Unit, General Hospital “George Papanikolaou”, 57010 Thessaloniki, Greece; tmdmgr@gmail.com (M.K.); lila.papalexandri@gmail.com (A.P.); tasoula.touloumenidou@gmail.com (T.T.); ioannamarilena@gmail.com (I.S.); 2 Second Propedeutic Department of Internal Medicine, Hippocration Hospital, Aristotle University of Thessaloniki, 54642 Thessaloniki, Greece; pascevan@auth.gr; 3 Children & Adolescent Hematology-Oncology Unit, Second Department of Pediatrics, School of Medicine, Aristotle University of Thessaloniki, 54124 Thessaloniki, Greece; hatzip@auth.gr (E.H.); atragian@auth.gr (A.T.)

**Keywords:** acute lymphoblastic leukemia, bulk RNA sequencing, multi-omics, single-cell RNA sequencing, spatial transcriptomics, targeted RNA sequencing

## Abstract

Acute Lymphoblastic Leukemia (ALL) is a highly heterogeneous hematological malignancy characterized by diverse genetic alterations. Advances in genomic and transcriptomic profiling have enabled refined ALL classification, improving clinical management and patient outcomes. This review provides a comparative evaluation of RNA sequencing methodologies for studying gene expression in ALL. Specifically, bulk RNA sequencing enables transcriptome-wide profiling at the population level, while targeted RNA sequencing provides enhanced sensitivity for detecting clinically relevant alterations. Moreover, single-cell RNA sequencing offers cellular-resolution analysis of leukemic heterogeneity and clonal architecture, whereas spatial transcriptomic analyses further reveal leukemic cell types within their microenvironment. Together, these transcriptomic methodologies have transformed the understanding of leukemia biology and enabled improved disease classification, risk stratification, and personalized medicine. Furthermore, integrating multi-omics approaches has the potential to reshape the clinical management of ALL by shifting treatment from broad chemotherapy to precision medicine. The future of transcriptomic profiling in ALL depends on the strategic integration of existing methodologies to achieve a comprehensive and clinically actionable understanding of leukemic biology.

## 1. Introduction

Acute Lymphoblastic Leukemia (ALL) is one of the most molecularly well-characterized hematological malignancies, especially in the pediatric setting. Despite remarkable improvements in survival, particularly among pediatric patients, ALL remains associated with substantial morbidity and mortality in adolescents, adults, and patients with relapsed or refractory disease. Extensive genomics and transcriptomics studies have revealed that ALL comprises multiple genetically and transcriptionally distinct subtypes, defined by chromosomal rearrangements, copy number alterations, and sequence mutations. These advances in genomic and transcriptomic profiling have enabled refined ALL classification and facilitated the development of precision medicine approaches, highlighting the increasing importance of comprehensive molecular profiling in guiding individualized treatment strategies [1,2].

**Figure 1:**
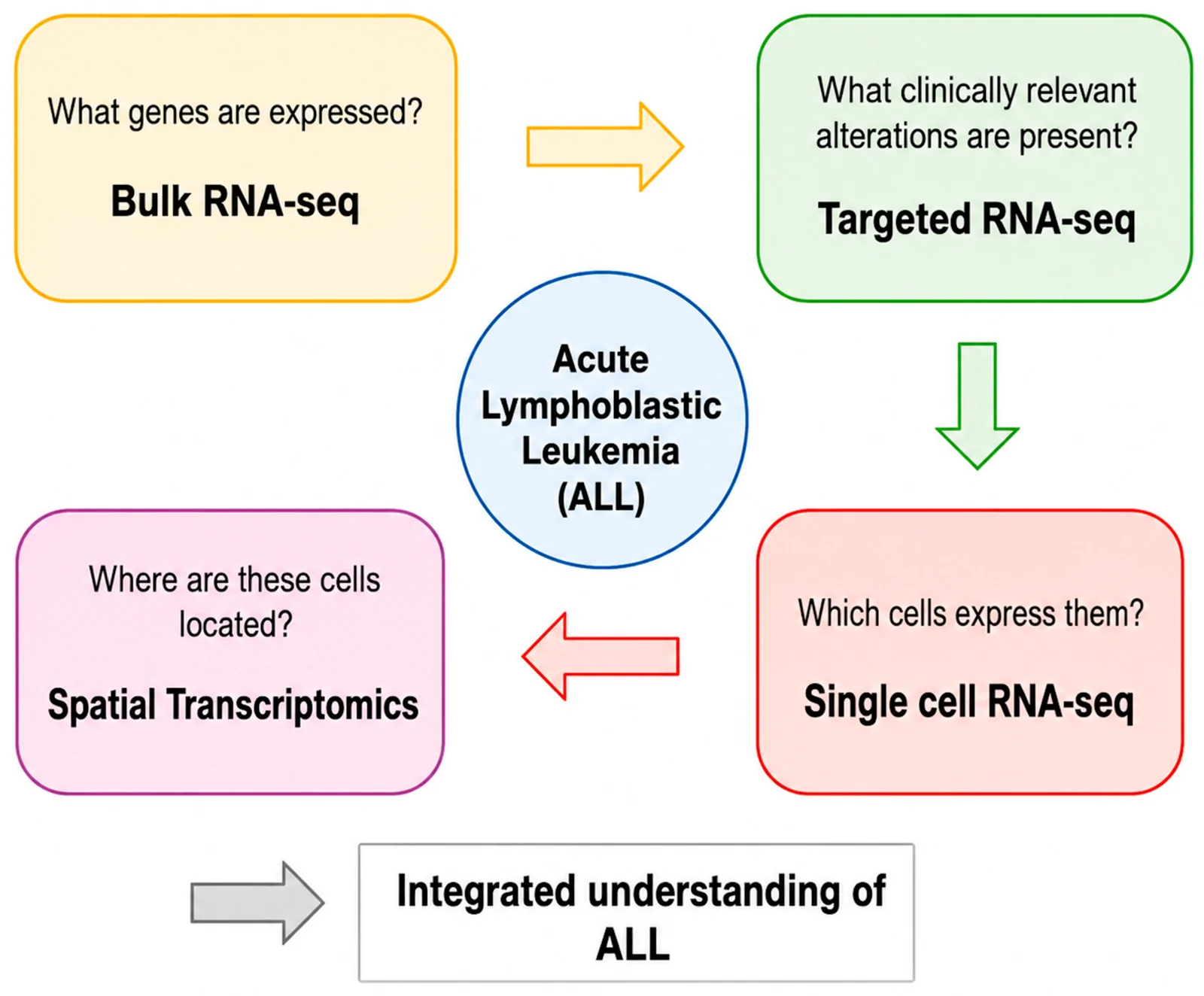
Evolution of transcriptomic technologies in ALL. Bulk RNA-seq enables transcriptome-wide profiling at the population level, whereas targeted RNA sequencing focuses on clinically relevant genomic alterations. Single-cell RNA-seq provides cellular-resolution analysis of leukemic heterogeneity and clonal architecture, while spatial transcriptomics preserves tissue context and cell–cell interactions within the bone marrow microenvironment. Together, these complementary approaches contribute to an increasingly comprehensive understanding of ALL biology. ALL: acute lymphoblastic leukemia, Bulk RNA-seq: Bulk Ribonucleic Acid Sequencing, RNA: Ribonucleic Acid, Single-cell RNA-seq: Single-cell RNA sequencing, Targeted RNA-seq: targeted RNA sequencing.

Advances in high-throughput sequencing technologies continue to unravel the genomic landscape of ALL, ranging from bulk population-level analyses to single-cell and spatially resolved approaches. Bulk RNA sequencing (bulk RNA-seq) has proven particularly valuable for detecting recurrent gene fusions, assigning expression-based subtypes, and performing transcriptome-wide classification across large pediatric and adult ALL cohorts. Recently, spatially resolved transcriptomic approaches have highlighted the cellular complexity of the ALL microenvironment [3,4,5]. Although a wide range of transcriptomic methodologies is currently employed to study gene expression in ALL, each approach offers distinct analytical strengths and limitations. A systematic technical comparison of these methodologies is therefore essential to guide their appropriate application in ALL research and clinical contexts (Figure 1).

## 2. Overview of Transcriptomic Technologies

ALL is a molecularly heterogeneous hematological malignancy driven by diverse genetic alterations that lead to oncogene dysregulation or the expression of chimeric fusion transcripts. Expression profiling has been successfully used in refining the molecular taxonomy of ALL by revealing genetic subtypes defined by diverse and complex molecular abnormalities with diagnostic and prognostic relevance. In parallel, recent advances in RNA sequencing (RNA-seq) technologies have facilitated the integration of transcriptomic profiling across a wide range of experimental and clinical settings. Although RNA-seq is a well-established technology for gene expression profiling in research settings, many challenges remain in translating it into clinical practice [2,6].

A wide range of transcriptomic methodologies are used in biomedical research, such as bulk RNA-seq, targeted RNA panels, and, more recently, single-cell RNA sequencing (scRNA-seq). Bulk RNA-seq is widely used for comprehensive quantification of gene expression at the population level, enabling massively parallel, in-depth gene expression profiling of diverse cell mixtures [7]. In contrast, targeted RNA sequencing (targeted RNA-seq) assays offer enhanced sensitivity for detecting therapeutically and prognostically significant fusions and other diagnostically significant sequence variants [8]. In recent years, scRNA-seq technologies have offered higher resolution by enabling transcriptomic profiling at the level of individual cells, thereby overcoming the limitations of bulk RNA-seq and allowing the identification of distinct cellular states [9]. Regardless of their methodological approaches, different transcriptomic techniques offer distinct insights into ALL. Bulk RNA-seq has remarkably advanced our understanding of ALL, enabling detailed gene expression profiling, the discovery of novel therapeutically relevant subtypes, and a population-level view of transcriptional deregulation. Dana-Farber Cancer Institute ALL Consortium Protocol 16-001 demonstrated that transcriptome-wide sequencing can be incorporated into the diagnostic workflow within a clinically relevant timeframe while maintaining high concordance with conventional molecular diagnostic methods. Importantly, RNA-seq identified additional clinically relevant fusion transcripts and molecular alterations that were not detected by routine diagnostic testing, highlighting its added value for comprehensive molecular classification and precision medicine in ALL [10]. These findings have subsequently been supported by independent clinical studies, which demonstrated high concordance between bulk RNA-seq and conventional diagnostic methods while identifying additional clinically relevant fusion transcripts that were not detected by routine molecular testing [11,12]. Complementary evidence supporting the clinical implementation of RNA-seq has also been reported for targeted RNA-seq [13]. This has also been shown by a recent UK single-center clinical study, in which the routine implementation of targeted RNA-seq improved molecular risk stratification, reclassified approximately 5% of patients into a high-risk category through the identification of cryptic fusion genes, and directly influenced therapeutic decision-making in 18% of cases, while also facilitating fusion-based minimal residual disease (MRD) assay design in patients lacking conventional Ig/T cell receptor (TCR) markers [14]. At a higher level of resolution, scRNA-seq has revealed previously unrecognized leukemic cell states, tissue heterogeneity, cellular interactions, and their functional states within complex biological samples [15]. Extending this approach to tissue architecture, spatial transcriptomic analyses further reveal leukemic cell types within their microenvironment, revealing their dysfunctions in detail and uncovering interactions with stromal niches that contribute to disease maintenance and progression [5].

## 3. Transcriptomics-Driven Molecular Classification of ALL

Currently, ALL is classified into genetic subtypes based on cytogenetically defined chromosomal aberrations of diagnostic and prognostic relevance. However, conventional cytogenetic and molecular methods, like karyotyping, in situ hybridization, and reverse transcription polymerase chain reaction (PCR), fail to identify sentinel alterations in 20–30% of ALL cases. High-resolution profiling of genetic alterations has transformed our understanding of the genetic basis of ALL and redefined its molecular taxonomy, revealing new molecular subtypes, dysregulated pathways, and therapeutic targets. These advances highlight the use of sequencing technologies to improve diagnostic accuracy and treatment outcomes, emphasizing the role of genomic data in advancing personalized treatments in ALL [16,17]. Over the last decade, the broad application of RNA-seq has enabled the discovery of novel molecular subtypes in ALL. A characteristic example is Philadelphia chromosome–like (Ph-like) ALL, a high-risk subset with a gene expression profile highly similar to that of BCR-ABL-positive ALL but lacking the BCR::ABL1 fusion. Ph-like ALL is a heterogeneous disease, mainly characterized by kinase-activating alterations with important therapeutic implications [18]. More recently, the incorporation of transcriptional profiling for molecular classification in ALL has shown improved risk assessment in BCR-ABL1–negative B-cell ALL (B-ALL), supporting RNA-seq utility in clinical practice for diagnosis of molecular subtypes [19].

Transcriptomic profiling has also demonstrated significant clinical value, uncovering therapeutically actionable alterations in ALL. BCR-ABL1-positive ALL is a high-risk disease subtype treated with tyrosine-kinase inhibitors (TKIs). Deep molecular profiling by RNA sequencing has discovered three transcriptomic subtypes of BCR-ABL1-positive ALL. Each subtype represents a maturation arrest at a stage of B-cell progenitor differentiation, indicating that treatment response and TKI efficacy depend on the differentiation stage at which the leukemia transforms [20]. Moreover, large-scale molecular profiling studies have enabled refined risk assessment and patient stratification, identifying novel fusion events that can aid advanced diagnostics and improved clinical management of patients [21]. The integration of transcriptomic classification into diagnostic routines can greatly facilitate advanced molecular subtyping of patients and improve risk assessment, thereby enabling more personalized treatment approaches.

In summary, transcriptomic profiling has significantly deepened our understanding of disease biology and established the foundation for personalized treatment approaches for patients with ALL. The application of RNA-seq to routine diagnostic workflows remains challenging due to the complexity of data processing and interpretation [8]. Even with the advances in molecular characterization of ALL, there are cost and health-economic considerations for the clinical use of sequencing technologies in broader clinical settings [22]. Substantial disparities remain in access to next-generation sequencing (NGS) infrastructure, bioinformatics expertise, and molecular diagnostic services across regions worldwide. These challenges are particularly evident in low- and middle-income countries, where limited resources may restrict the implementation of advanced transcriptomic technologies [23]. Furthermore, variations in reimbursement policies and healthcare funding mechanisms may also influence access to sequencing-based diagnostics, even among high-income countries [24]. In conclusion, these clinical and practical considerations highlight the need to develop robust benchmarking frameworks and to expand access to NGS in hematology units worldwide.

## 4. Bulk RNA-Seq

### 4.1. Technical Principles

**Figure 2:**
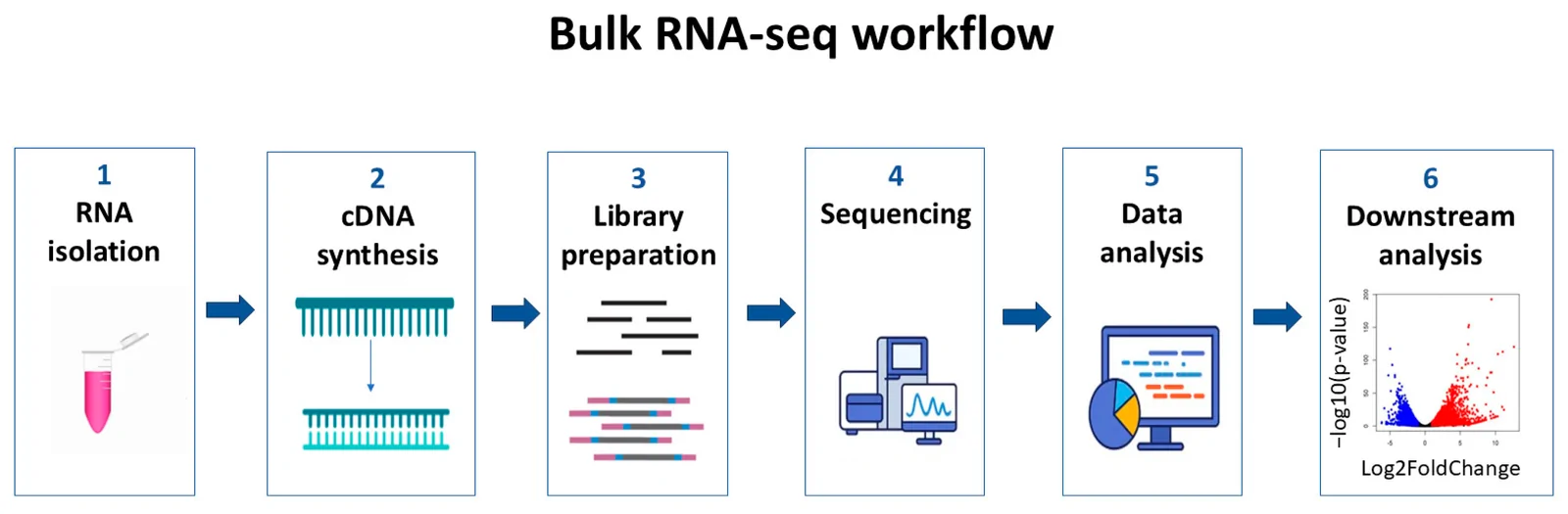
Schematic overview of a typical bulk RNA-seq workflow. Total RNA is isolated from the biological sample, followed by reverse transcription to cDNA, library preparation through fragmentation and adapter ligation, high-throughput sequencing, and downstream bioinformatic analysis. The resulting transcriptomic data enable gene expression quantification, differential expression analysis, fusion transcript detection, transcript isoform analysis, and pathway enrichment analysis. Bulk RNA-seq: Bulk Ribonucleic Acid Sequencing, cDNA: complementary deoxyribonucleic acid, Log2FoldChange: Logarithm2 fold change, −log10(*p*-value): −10logarithm *p*-value, RNA: ribonucleic acid. Figure was created using Microsoft PowerPoint (Microsoft Office Professional Plus 2021, Microsoft Corporation, Redmond, WA, USA).

To explore the functional importance of genes of interest, researchers have turned to whole-transcriptome analysis. Bulk RNA-seq is a widely used technique that enables simultaneous measurement of the expression levels of thousands of genes in a mixture of cells [25]. Several NGS platforms are available, but they differ in detection technologies and data outputs. For instance, Illumina platforms use fluorescence to detect bases, while Ion Torrent platforms measure pH changes to identify bases. Despite differences, all NGS platforms require converting the source nucleic acid material into standard libraries suitable for loading onto a sequencing instrument (Figure 2) [26]. A typical experiment requires the isolation of RNA from samples, the preparation of complementary DNA (cDNA) libraries, sequencing, and finally, bioinformatic analysis. Library preparation workflows typically include mRNA fragmentation, first-strand cDNA synthesis, second-strand cDNA synthesis, adapter ligation, and DNA fragment enrichment. Bulk RNA-seq methods are sensitive to RNA input amount, retention of strand specificity, and the presence of multiple RNA species that differ in size, sequence, structural features, and abundance. The choice of the RNA-seq library preparation method depends on several factors, including cost, RNA quality, and RNA input [27].

Data analysis of raw data generated after sequencing involves several steps. Output from NGS is typically in the form of FASTQ files, which contain sequencing reads. These reads must be mapped or aligned to the sample’s reference genome, and then the counts for each transcript can be estimated. This can then be followed by an analysis of differentially expressed genes and plotting of gene expression data. All these steps require robust bioinformatics pipelines. The challenge for researchers is to improve data analysis methods so that the vast amount of generated data can be managed efficiently [25]. Almost all steps of the various library preparation workflows and bioinformatic pipelines introduce technical biases, especially in RNA-seq, which is more challenging than DNA sequencing. The main technical biases are: (1) PCR amplification bias leading to the uneven amplification of cDNA molecules; (2) sequencing composition bias where GC- or AT-rich regions can be under-represented; (3) read depth and coverage, which can affect the detection of lowly expressed genes and the accuracy of quantification across the transcriptome; and (4) alignment and mapping errors that can lead to incorrect transcript assembly or gene expression estimation [28]. Each of these biases requires specific strategies for data processing and analysis.

### 4.2. Applications of Bulk RNA-Seq in ALL

Subtyping-defining chromosomal alterations in ALL are heterogeneous and sometimes cannot be detected with conventional cytogenetics. RNA-seq in large patient cohorts enables the identification of clinically relevant gene fusions and sequencing mutations, as well as the accurate identification of novel molecular subtypes with prognostic and/or therapeutic significance (Table 1). Additionally, this technique can identify clonal mutations in diagnostic samples that may have prognostic and therapeutic impact [10]. The classification of ALL based on gene expression patterns in larger ALL cohorts helped identify rare subtypes. In a recent study, 10 novel subtypes of T-cell ALL (T-ALL) were identified, each characterized by distinct gene mutation profiles and dysregulated expression signatures of leukemogenic factors, improving the previous classification of T-ALL [4].

Even a single approach, such as bulk RNA-seq, can identify many additional molecular features in ALL that have the potential to be clinically actionable. Approximately 25–30% of newly diagnosed B-cell precursor ALL cases are initially classified as B-other (or B-ALL, not otherwise specified), as they lack the recurrent cytogenetic abnormalities routinely assessed in diagnostic laboratories. Rather than representing a single biological entity, B-other ALL comprises a heterogeneous group of genetically distinct leukemias that remained unclassified using conventional cytogenetic and molecular approaches. Transcriptome-wide RNA sequencing has substantially refined the molecular classification of these cases by identifying recurrent gene fusions, expression signatures, and cryptic genomic alterations, enabling reclassification into recently recognized molecular subtypes such as DUX4-rearranged, ZNF384-rearranged, MEF2D-rearranged, PAX5-altered, NUTM1-rearranged, ETV6::RUNX1-like, and Ph-like ALL. Among these, Ph-like ALL represents a clinically important high-risk subgroup characterized by genomic alterations that activate kinase and cytokine receptor signaling, thereby complicating diagnostic molecular characterization. The incorporation of RNA-seq into diagnostic workflows has significantly improved the molecular classification of ALL, facilitating accurate diagnosis, risk stratification, and selection of patients who may benefit from targeted therapies, including tyrosine kinase or JAK inhibitors [1,13,29,30]. Implementation of RNA-seq in clinical diagnostics has shown significant clinical impact, especially in patients not classified into an established subtype, specifically in B-other (a subtype of B-ALL defined by the absence of common, routine genetic alterations) or T-ALL [3]. Additionally, there are high-risk groups, such as patients with Ph-like ALL harboring a variety of genomic alterations that activate kinase and cytokine receptor signaling, thereby complicating diagnostic molecular characterization. RNA-seq enabled real-time identification of Ph-like ALL and targetable kinase and cytokine receptor alterations in B-ALL, suitable for treatment stratification [29].

### 4.3. Limitations of Bulk RNA-Seq in the Clinical Context

Bulk RNA-seq has become a valuable and extensively used tool in understanding cancer biology. Numerous RNA-seq approaches have been developed and validated across many major tumor types, but only a few of them have been successfully translated into clinical practice. The main reason seems to be the low reproducibility due to sampling bias resulting from tumor heterogeneity. Bulk RNA methods include multiple RNA species and detect multiple forms of genomic alterations. Moreover, bulk RNA-seq measures the average signal across all cells in a sample and thus may fail to capture cellular diversity, leading to misinterpretations of the molecular landscape. Additionally, there are significant technical challenges, such as the amount and quality of RNA, especially in diagnostic samples, and the variability of pipelines, including different NGS platforms and a variety of library preparation protocols [25,27].

The rapid development of transcriptomic technologies for B-ALL has led to ongoing discussions as to how health care systems can provide these diagnostic services to patients. The main clinical benefit of bulk RNA-seq is its ability to detect and classify patients into B-ALL genomic subtypes that might otherwise go unnoticed with conventional cytogenetic and molecular genetic techniques. However, there are additional clinical and practical factors to consider. In particular, bulk RNA-seq has a longer turnaround time and higher costs. Therefore, standardized clinical algorithms, including guidelines for variant interpretation and optimized bioinformatic pipelines, are needed to overcome limitations and enable the clinical implementation of bulk RNA-seq [22,31,32]. Consequently, more focused transcriptomic approaches have been developed to facilitate high-throughput, low-cost molecular profiling, leading to the increasing use of targeted RNA-seq in diagnostic settings.

## 5. Targeted RNA-Seq Panels

### 5.1. Technical Design and Analytical Performance

Targeted RNA-seq focuses sequencing on genes of interest rather than the whole transcriptome. Targeted panels are often practical in clinical settings for detecting clinically informative genetic alterations, such as gene fusions, point mutations, short indels, alternative splicing events, and expression levels. Like other NGS methods, the targeted RNA-seq workflow includes four steps: sample preparation, library preparation, sequencing, and data analysis. There are two main approaches to library preparation: amplicon-based and hybridization-capture methods. Amplicon sequencing uses PCR primers to amplify predefined genomic regions. Hybridization capture sequencing enriches target regions using sequence-specific probes that hybridize to DNA fragments of interest. Those two methods differ in sample input requirements, workflow complexity, number of targets per panel, sensitivity, turnaround time, cost, and overall efficiency. Targeted RNA-seq focuses sequencing depth on specific target genes and thus can detect lowly expressed transcripts and rare variants that are often missed or buried in the noise of bulk RNA-seq. Additionally, targeted RNA-seq requires less input material and can be performed on degraded samples. Analysis can be more feasible because the amount of data is more manageable than with whole-transcriptome approaches [8,33]. Unlike conventional amplicon-based approaches requiring prior knowledge of both fusion partners, Anchored Multiplex PCR (AMP) enables unbiased detection of known and novel fusion partners using a single gene-specific primer, making it particularly suitable for identifying promiscuous fusion genes frequently encountered in ALL. Several clinically validated targeted RNA-seq assays for leukemia, including AMP-based panels, exploit this strategy to improve fusion detection in routine molecular diagnostics [34]. The analytical performance of targeted RNA-seq has not been extensively described in diagnostic samples. In fusion events, a targeted RNA-seq approach is a reliable tool for detecting targetable gene fusions in clinical diagnostics. This RNA-based technique is more efficient and sensitive for fusion detection than DNA-based approaches, requiring less coverage and enabling more effective filtering of false-positive fusion events using robust bioinformatics tools for short-read alignment. However, there are some limitations, including the detection of somatic mutations. Although most of the mutations identified at the DNA level can be detected in RNA-seq data, the nonsense mutations were rarely detected [33,35]. Some of the limitations of this technique might be resolved with the optimization of the panel design and the bioinformatics pipelines. On the other hand, because some limitations have a biological explanation, RNA-seq should not replace the analysis of genomic DNA but should be used as a complementary technique to increase the molecular characterization of hematologic malignancies at diagnosis.

### 5.2. Clinical Applications in ALL

In hematological malignancies, the goal of diagnostic procedures is not only to accurately classify the patient’s disease but also to identify biomarkers of prognostic or predictive value. Targeted RNA-seq is increasingly used in clinical molecular characterization of ALL, as it efficiently detects multiple genetic alterations in a single test. In ALL, this technique has demonstrated high analytical performance in detecting subtype-defining fusion transcripts, intragenic deletions, and aberrant expression profiles, enabling accurate molecular classification and improved risk stratification. In more detail, apart from the well-known subtypes based on the presence of BCR-ABL1, rearrangements of KMT2A (MLL), TCF3 (E2A)-PBX1 and ETV6-RUNX1, targeted RNA-seq can be used to identify some other molecular markers of ALL, defined by fusions and mutations, like PAX5 P80R alteration, IGH-CRLF2 fusion, intragenic ERG deletions, IKZF1 deletions, and subgroups defined by expression profiles, like DUX4-rearrangements [13,36]. Integrating targeted RNA-seq into routine diagnostics demonstrates its practical applicability in clinical settings as a reliable tool for detecting the growing diversity of targetable gene fusions, recurrent mutations with clinical significance, and identifying transcriptional profiles associated with clinically relevant entities.

### 5.3. Clinical Implementation and Routine Diagnostics

Targeted RNA panels have demonstrated high sensitivity and specificity for detecting clinically informative genetic alterations, and a range of commercially available assays is being established in clinical diagnostic laboratories today. As NGS technologies emerge and evolve, strict validation and standardization of these approaches are imperative for implementation in clinical diagnostic settings to ensure analytical accuracy and consistency across laboratories. The analysis of the transcriptome in routine diagnostic procedures is technically challenging, and many considerations must be taken into account. The choice of sequencing method depends on the size of the panel, the specific needs of the regions of interest, the required sensitivity, the required test turnaround time, the availability of bioinformatics support, and the technical expertise of clinical laboratories. All of the above highlight the need for established guidelines for targeted panels to assist clinical laboratories with the validation and ongoing monitoring of NGS testing [34,37]. Current international guidelines acknowledge the growing diagnostic value of RNA-based assays, particularly for the identification of cryptic gene fusions and Ph-like ALL, while emphasizing the need for standardized validation and quality assurance before widespread clinical adoption [38]. Despite advances in bulk and targeted RNA-seq, these approaches remain limited in resolving cellular heterogeneity, highlighting the emerging role of scRNA-seq technologies.

## 6. Single-Cell Transcriptomics

### 6.1. Technical Overview and Analytical Platforms

ALL is a highly heterogeneous malignancy. The ability to decode tumor complexity is transforming the understanding of cancer evolution, response to therapy, and the ability of tumors to adapt to novel conditions. Population-level approaches (Bulk RNA-seq) fail to fully resolve this complexity, as they tend to mask the unique expression patterns of smaller but clinically relevant cell populations. ScRNA-seq has been extensively used to characterize the tumor microenvironment, to determine the clonal architecture, to subtype leukemia, and to reveal drug response and resistance [39]. The rapid advancement of single-cell technologies, including other omics, across cancer research will contribute to precision medicine in clinical settings [40].

#### 6.1.1. Platforms and Data Generation

**Figure 3:**
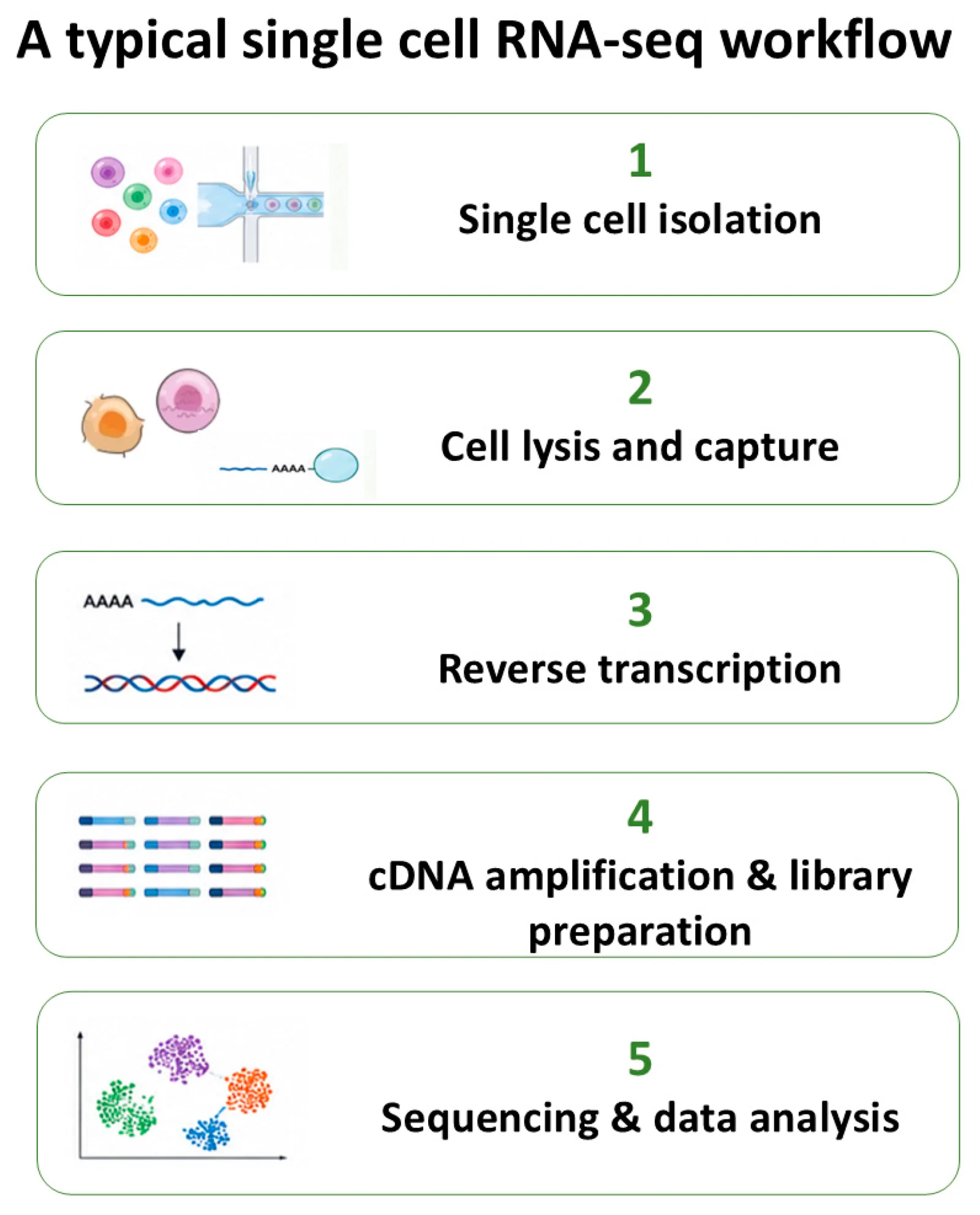
Schematic overview of a typical scRNA-seq workflow. Individual cells are isolated and lysed before reverse transcription and cDNA amplification. Sequencing libraries are subsequently prepared and subjected to high-throughput sequencing, followed by computational analyses including quality control, cell clustering, cell-type annotation, differential gene expression, trajectory inference, and characterization of cellular heterogeneity. cDNA: complementary deoxyribonucleic acid, RNA: ribonucleic acid, ScRNA-seq: single-cell RNA sequencing. Figure was created using Microsoft PowerPoint (Microsoft Office Professional Plus 2021, Microsoft Corporation, Redmond, WA, USA).

A typical scRNA-seq protocol includes several steps: (1) single-cell isolation, (2) cell lysis, (3) reverse transcription, (4) cDNA amplification, (5) library preparation, (6) sequencing, and (7) data analysis (Figure 3). The two main methods for library preparation in scRNA-seq are plate-based methods and droplet-based methods. Plate-based methods (Smart-seq) offer high sensitivity, allow full-length sequencing of transcripts, and improve the detection of splice variants. In contrast, droplet-based methods (10xGenomics, Drop-seq) offer high-throughput profiling of thousands of cells, enabling the detection of rare cells at lower cost [41].

#### 6.1.2. Preprocessing and Quality Control

The preprocessing of scRNA-seq data involves multiple steps, including quality control, filtering, and normalization, which can significantly influence downstream analyses such as cell-type clustering and trajectory inference. Filtering thresholds aim to exclude low transcript counts or cells with high mitochondrial expression. Normalization methods are necessary to remove cell-specific bias and account for differences in sequencing depth and technical variability, which can affect downstream applications. However, there is no consensus approach for preprocessing scRNA-seq data, and diverse techniques can be a major source of variability across studies [41].

#### 6.1.3. Core Analytical Framework

After normalization, the main computational steps for scRNA-seq data analysis aim to reduce dimensionality, identify cell populations, and determine biological differences. The first step is selecting highly variable genes to capture the most informative features, followed by scaling and dimensionality reduction using an unsupervised linear method, principal component analysis. The next step is clustering, in which cells are grouped based on their gene-expression similarity using graph-based algorithms. Uniform manifold approximation and projection and t-distributed stochastic neighbor embedding are two non-linear dimensionality reduction techniques applied to visualize cell clusters. Even though these techniques are widely used, clustering remains sensitive to parameter choices and preprocessing, highlighting the need for careful interpretation of analytical outputs [41,42].

#### 6.1.4. Data Integration and Analytical Challenges

In scRNA-seq experiments, cells from a single condition are typically captured and sequenced. ScRNA-seq data is often compiled from multiple experiments with differences in sample processing, sequencing platforms, and experimental conditions. These differences lead to batch-specific systematic variations in the data and can confound biological variations in interest during data integration. Additionally, single-cell data are sensitive to “dropout” events where transcripts present in a cell are not detected. To address these issues, various batch removal methods are used to align similar cell types across samples while preserving biological differences, such as Harmony, LIGER, and Seurat 3. All three methods have advantages and limitations in their approach, speed, and handling of biological variation. For example, LIGER and Seurat often perform stronger batch removal, which can lead to overcorrection, merging different cell types, or obscuring true biological differences [41,43,44].

#### 6.1.5. Advanced Analytical Approaches

Over the past few years, advanced analytical approaches in scRNA-seq data have enabled the reconstruction of dynamic biological processes and intercellular interactions. Researchers have developed several experimental and computational methods that are focused on studying the dynamics of biological processes using time-series scRNA-seq data. Trajectory inference methods order cells along a continuum to reconstruct dynamic biological processes, such as cell differentiation, development, or disease progression [45]. In parallel, cell–cell communication analyses allow researchers to build signaling networks determining how cells interact by analyzing ligand-receptor pairs expressed by different cell populations [46]. All these integrative frameworks have resulted in great progress in our understanding of cancer pathogenesis, disease progression, and drug resistance [47].

### 6.2. Key Applications in ALL

The most significant impact of scRNA-seq on ALL research is the detailed dissection of leukemic heterogeneity. Unlike bulk RNA-seq, which measures the average gene expression across mixed cellular populations, scRNA-seq reveals transcriptional, functional, and developmental complexity within ALL cell populations (Table 2). To illustrate, a large-scale pediatric study defined the cellular states that characterized treatment failure in NOTCH1-Mutant relapsed early T-cell precursor ALL (ETP-ALL) treated with a Notch inhibitor by revealing complex interactions among signaling programs, cellular plasticity, and immune programs that characterize ETP-ALL [48]. Single-cell transcriptomic analyses have also substantially improved our understanding of leukemia biology by placing leukemic blasts within the context of normal hematopoietic development. Comparison with reference developmental atlases has demonstrated that genetically distinct ALL subtypes are characterized by specific developmental arrest states and differentiation trajectories. Furthermore, developmental programs identified by scRNA-seq have been associated with treatment response, relapse risk, and lineage plasticity, highlighting their potential biological and clinical significance [49,50]. For example, in infant B-cell ALL, researchers identified a unique developmental program associated with KMT2A-rearranged disease, emphasizing that subtype-specific transcriptional identities can be resolved only at single-cell resolution [51]. Similarly, single-cell transcriptomic studies of hyperdiploid ALL have shown that most chromosomal gains are acquired early during leukemogenesis and remain remarkably stable throughout clonal evolution, supporting a punctuated model of disease development and providing new insights into the developmental origins of this ALL subtype [52]. Additionally, scRNA-seq approaches in rare leukemia subgroups such as mixed phenotype acute leukemia (MPAL) supported the existence of leukemia-enriched transcriptional signatures that distinguish malignant from non-malignant pediatric hematopoietic populations [53].

ScRNA-seq has improved understanding of the leukemia microenvironment, uncovering critical interactions between leukemic blasts and their surrounding cells. Basic research in the tumor microenvironment highlighted the importance of resolving both malignant and non-malignant cellular compartments [49]. Single-cell studies in pediatric T-ALL identify a subgroup characterized by a remodeled immune microenvironment, which is associated with adverse clinical outcomes, suggesting that immune suppression and altered stromal signaling may influence treatment response [54]. Similarly, single-cell genomic analysis has demonstrated that T-ALL develops through stepwise acquisition of mutations across hierarchically related subclones, with evidence that leukemogenic lesions may originate in early multipotent progenitor cells and that canonical driver events such as NOTCH1 mutations often occur relatively late during disease evolution [55].

Single-cell transcriptomics offers opportunities for more precise MRD detection and risk stratification than conventional bulk assays. A scRNA-seq study highlighted the clinical potential by revealing that persistent leukemic blast signatures and MRD-associated immune alterations can be captured simultaneously through single-cell transcriptomic profiling in pediatric T-ALL [56]. Additionally, in B-ALL, researchers reported that marked immunometabolic dysregulation across B-ALL subtypes revealed subtype-specific signaling dependencies that may serve as potential therapeutic targets [57]. However, the use of scRNA-seq for MRD assessment remains investigational and has not been incorporated into routine clinical practice or current ALL management guidelines.

**Table 2:** Representative studies employing scRNA-seq in ALL.

Reference, First Author (Year)	Study Design	Age Group	ALL Subtype	Single-Cell Technology	Key Findings
Mumme (2025) [58]	Development of a pediatric single-cell leukemia atlas	Pediatric	Multiple ALL subtypes/MPAL	scRNA-seq (10x Genomics)	Generated a comprehensive pediatric leukemia cell atlas and identified leukemia-enriched transcriptional signatures useful for disease classification
Khabirova (2022) [51]	Single-cell characterization of infant leukemia	Infant	KMT2A-rearranged B-ALL	scRNA-seq + bulk transcriptomic integration	Demonstrated that KMT2A-rearranged infant B-ALL exhibits an early lymphocyte precursor-like developmental state with hybrid myeloid–lymphoid features
De Bie (2018) [55]	Clonal evolution and mutation ordering study	Pediatric/young adult	T-ALL	Single-cell sequencing	Reconstructed the order of mutation acquisition and showed that NOTCH1 alterations are frequently secondary events in T-ALL evolution
Ferrao Blanco (2025) [5]	Bone marrow microenvironment analysis	Pediatric B-ALL	B-ALL	scRNA-seq + spatial transcriptomics	Identified distinct stromal populations supporting leukemic survival and chemoresistance within the marrow niche
Sun (2025) [57]	Immunometabolic profiling	B-ALL	B-ALL	scRNA-seq	Revealed subtype-specific metabolic and immune programs and highlighted potential therapeutic targets
Bhasin (2023) [56]	MRD-associated immune landscape analysis	Pediatric	T-ALL	scRNA-seq	Defined blast-associated transcriptional programs and immune microenvironment changes linked to MRD
Wiggers (2025) [54]	Tumor microenvironment study	Pediatric	T-ALL	scRNA-seq	Immune microenvironment remodeling is associated with adverse clinical outcomes in pediatric T-ALL

ALL: acute lymphoblastic leukemia, MPAL: mixed-phenotype acute leukemia, ScRNA-seq: single-cell ribonucleic acid sequencing.

### 6.3. Limitations

Despite its contribution to the study of ALL, scRNA sequencing remains associated with several important methodological limitations. ScRNA-seq data are characterized by high technical noise, dropout events, low sensitivity in detecting low-abundance transcripts, and variability across analytical pipelines. The dissociation of tissues into single-cell suspensions inevitably disrupts the native spatial organization of leukemic and non-leukemic cells, resulting in the loss of spatial information and introducing cell-type bias. Additionally, the relatively high cost, computational burden, and lack of standardization in data processing further restrict its widespread clinical implementation [40,41,47]. Consequently, although scRNA-seq provides unprecedented resolution of cellular heterogeneity and clonal dynamics, thereby overcoming some of these limitations, spatial transcriptomic technologies have emerged as a critical extension of RNA-based profiling by combining transcriptomic resolution with the preservation of tissue architecture.

## 7. Spatially Resolved Transcriptomics (SRT)

Although scRNA-seq is a powerful tool for investigating cellular heterogeneity, most scRNA-seq approaches involve isolating cells from their original positions, thereby losing spatial information. SRT is a new technology that aims to determine gene expression profiles while preserving spatial tissue context [59].

### 7.1. Technical Overview

Based on how the data are obtained, SRT methods can be broadly classified into imaging-based and sequencing-based methods. Imaging-based methods, such as in situ hybridization or in situ sequencing, use fluorescent signals and their intensities to decode the targeted gene and its abundance. Sequencing-based methods rely on spatial barcodes on an array to restore the spatial position of the targeted gene in the tissue and then use next-generation sequencing to determine its expression level [60].

Data analysis in SRT can be challenging due to increased data volume and the complexity of integrating gene expression with spatial imaging. General pipelines for SRT data analysis include computational approaches and integration methods (with bulk or single-cell RNA-seq data), followed by analytical methods for identifying localized gene expression patterns, spatial decomposition, gene imputation, and cell–cell communication [60]. When designing an SRT experiment, there are several essential considerations in choosing a suitable spatial technology. These considerations include: the biological question to be answered; the type of tissue available for the study; the quality of RNA; tissue size; the number of genes profiled; spatial resolution (especially if scRNA resolution is required); and the number of genes to be analyzed [61].

#### Bone Marrow Challenges

While SRT technologies were initially developed for the study of solid tumors, their application to hematological malignancies is challenging due to the diffuse, highly dynamic nature of the bone marrow microenvironment, which lacks the well-defined architectural features found in solid tumor tissues. Spatial transcriptomic approaches have highlighted the importance of the bone marrow microenvironment, illustrating that hematopoietic and leukemic cells are organized within distinct spatial niches that regulate cellular function and interactions, thereby requiring adapted spatial approaches and careful interpretation of spatially resolved data [62].

### 7.2. Applications of SRT in Hematological Malignancies

Although the application of SRT technologies in ALL remains relatively limited, emerging studies have revealed the cellular complexity of the bone marrow microenvironment, identifying distinct specialized stromal niches that ALL cells can interact with. These findings emphasized that stromal cells actively participate in leukemic cell survival, immune modulation, and disease maintenance rather than serving merely as structural support [5].

Beyond ALL, spatial profiling studies in other hematologic malignancies already demonstrate the method’s potential to uncover bone marrow niches, immune organization, and microenvironmental interactions that cannot be captured by single-cell transcriptomics. Spatial transcriptomic analyses of myelofibrosis have revealed that bone marrow demonstrates intense spatial reorganization, inflammatory niches, and pathological stromal areas [63]. Similarly, multidimensional spatial analyses in pediatric acute myeloid leukemia identified distinct immune phenotypes and spatially organized immune aggregates associated with leukemic progression and local immune regulation [64]. Collectively, such studies demonstrate that SRT and related spatial omics approaches can uncover biologically relevant cellular interactions that remain obscured in dissociative single-cell sequencing workflows.

Recent multimodal spatial omics approaches, including spatial proteomic profiling studies in acute myeloid leukemia, further suggest that integrating transcriptomic and proteomic spatial information may provide a more comprehensive characterization of the leukemic microenvironment and its immunological organization [65].

### 7.3. Limitations and Future Perspectives

Broad application of SRT in hematological malignancies is restricted due to several technical and analytical limitations. The spatial resolution of current platforms often involves trade-offs between transcriptomic coverage and spatial resolution, with many approaches lacking true single-cell resolution. Unlike solid tumors, bone marrow samples present unique challenges due to the structural fragility, cellular heterogeneity, and complex architecture of hematopoietic tissues [62]. An additional challenge unique to bone marrow trephine biopsies is the requirement for decalcification before sectioning. Conventional acid-based decalcification protocols can substantially compromise RNA integrity, reduce transcript recovery, and affect the performance of sequencing-based spatial transcriptomic assays. Current evidence supports the use of ethylenediaminetetraacetic acid (EDTA)-based decalcification, together with standardized tissue processing workflows, to better preserve nucleic acids while maintaining tissue morphology. Alternative approaches, including optimized mild decalcification protocols and the use of fresh-frozen specimens when feasible, may further improve RNA preservation and facilitate the implementation of spatial transcriptomics in bone marrow samples [62,66]. SRT data often suffer from high technical noise and technical bias, spatial batch effects, and a lack of standardized pipelines for proper handling of spatial information. Additionally, the high cost of spatial assays, substantial computational requirements, and limited standardization of analytical workflows also remain significant barriers to routine clinical implementation [61].

Nevertheless, continued improvements in spatial resolution are expected to further enhance the applicability of spatial omics technologies in hematologic malignancies and may ultimately facilitate their incorporation into precision hematology [67].

## 8. Comparative Analysis

While bulk RNA-seq, targeted RNA-seq, scRNA-seq, and SRT all rely on transcriptomic profiling, each methodology provides distinct layers of information and is associated with unique advantages, limitations, and clinical applications (Table 3). These transcriptomic technologies should be considered complementary tools that collectively contribute to a more comprehensive understanding of leukemic biology, clonal architecture, tumor microenvironment interactions, and therapeutic response.

### 8.1. Resolution and Biological Insight

Comparative analyses of transcriptomic methodologies have highlighted that increasing transcriptomic resolution is accompanied by greater analytical complexity, computational burden, and data dimensionality, while simultaneously enabling more refined characterization of cellular heterogeneity and tissue organization (Table 4). Bulk RNA-seq provides transcriptome-wide profiling at the population level and has significantly contributed to the molecular classification of ALL. This is a powerful technology that captures a complete snapshot of the transcriptome, accurately measures gene and transcript abundance, and identifies known and novel transcriptome features. However, because bulk RNA-seq averages gene expression across heterogeneous cellular populations, it cannot resolve rare leukemic subclones or transcriptionally distinct cellular states. In contrast, targeted RNA-seq “sacrifices” transcriptome-wide coverage in favor of increased sensitivity in detecting predefined, clinically relevant targets, such as fusion transcripts and recurrent leukemia-associated alterations. This method focuses on analyzing specific RNA transcripts rather than the entire transcriptome and provides highly sensitive data, enabling the detection of rare mutations and low-abundance transcripts. ScRNA-seq further expanded transcriptomic resolution by enabling the characterization of individual leukemic cells, thereby uncovering cellular heterogeneity, developmental hierarchies, and rare resistant populations that remain obscured in bulk analyses. ScRNA-seq analysis has revolutionized leukemia research by revealing cell types, pathways, and cellular interactions that play a critical role in malignant cell progression and response to therapy. Although scRNA-seq is a powerful stand-alone tool for characterizing the complex bone marrow microenvironment, it still has the limitation of tissue dissociation and, therefore, the loss of spatial context. Spatial transcriptomic sequencing addresses this limitation by preserving tissue architecture and enabling the localization of malignant, immune, and stromal populations within the bone marrow niche. This advanced technique maps RNA activity and reveals the identity, function, and position of cells within intact tissue, allowing researchers to observe exactly which genes are active and where those cells are located in their native microenvironment [68].

### 8.2. Clinical Applicability and Translational Utility

While all RNA sequencing methodologies contribute valuable biological information in ALL, their clinical applicability differs substantially in terms of analytical complexity, scalability, cost, and diagnostic utility. The primary clinical applications of bulk RNA-seq in ALL include identification of disease structural variants, molecular classification, and wide transcriptional profiling. Although bulk RNA-seq offers high scalability and robust reproducibility in large sample cohorts, it fails to address cell heterogeneity [4,10]. In parallel, targeted RNA-seq is the most clinically applicable method in ALL as it can accurately identify leukemia-driven fusion genes and cryptic structural variations, expression profiles required for subtype classification, and monitoring MRD. Targeted RNA panels demonstrate their practical applicability by reducing sequencing costs, turnaround time, and computational complexity compared to other sequencing methods [13,36]. On the other hand, scRNA-seq has transformed the understanding of ALL biology by resolving tumor heterogeneity and clonal evolution, paving the way for a new era of personalized medicine. Although highly effective at the investigational level, scRNA-seq has limited clinical integration due to high cost, lack of standardization, and computational burden [69]. Lastly, SRT is, for now, a growing translational research tool for decoding the complex bone marrow microenvironment, mapping relapse mechanisms, and evaluating responses to immunotherapies [59]. Consequently, rather than replacing one another, these methodologies are likely to play complementary roles within future precision oncology workflows, combining broad molecular profiling, targeted clinical diagnostics, cellular-resolution analysis, and spatial characterization of the leukemic microenvironment.

### 8.3. Technical Complexity and Limitations

Increasing transcriptomic resolution across RNA sequencing methodologies is accompanied by progressively greater technical, computational, and analytical complexity. While bulk and targeted RNA-seq remain the most cost-effective methods for high-throughput sequencing, scRNA-seq and SRT have relatively higher costs due to significant technical complexity, high-cost reagents, and the requirement of extensive computational power. Single-cell and spatial approaches generate substantially higher-dimensional datasets that require advanced computational frameworks and remain more vulnerable to technical variability. In contrast, bulk and targeted RNA-seq generally benefit from more standardized workflows and lower analytical complexity. In hematologic malignancies, sample requirements and processing pose additional challenges, as single-cell and spatial approaches depend on preserving cellular viability or tissue architecture, whereas bulk and targeted RNA-seq can often be performed on less demanding specimen types. Differences in sample requirements also contribute to variations in clinical feasibility, with bulk and targeted RNA-seq being more readily applicable to routine diagnostic specimens than single-cell or spatial transcriptomic methodologies [28,41,61].

### 8.4. Complementarity and Future Perspectives

No single RNA sequencing methodology can fully capture the biological complexity of ALL. Bulk RNA-seq provides transcriptome-wide profiling at the population level, while targeted RNA-seq focuses on detecting clinically relevant alterations. On the other hand, scRNA-seq has become one of the most popular genomic tools for dissecting transcriptome heterogeneity, though it is limited by the loss of spatial information. Moreover, SRT preserves the spatial context of tissues and provides novel insights into the tumor microenvironment. Bulk RNA-seq and scRNA-seq complement each other perfectly because their strengths offset each other’s limitations. Bulk RNA-seq is frequently used to validate single-cell findings in larger clinical samples. Additionally, computational tools for spatial technologies use single-cell data to map specific cell types to precise tissue coordinates and to discover how microenvironments influence gene expression and cellular state [70].

The future of ALL transcriptomics depends on transitioning from bulk RNA profiling to high-resolution, multi-omics, and machine-learning-driven single-cell and spatial analyses. This evolution promises to redefine clinical diagnosis, reveal hidden molecular subtypes, and profile the leukemia microenvironment with unprecedented precision. Deep learning algorithms are being actively trained on vast transcriptome datasets to automate the diagnosis and subclassification of ALL. These multi-omic approaches aim to drive personalized medicine, facilitating precise risk stratification, the discovery of targeted therapies, and highly sensitive monitoring of MRD [40,67].

Future advances in ALL research are likely to arise not from the replacement of existing methodologies, but from their strategic integration across multiple levels of biological organization.

### 8.5. Short-Read Versus Long-Read RNA Sequencing

Despite its widespread utility, short-read RNA-seq is fundamentally constrained by its limited read length. Short-read RNA-seq workflows require the fragmentation of mRNA molecules before sequencing, and cannot sequence intact transcript isoforms directly. Long-read RNA-seq is a sequencing technique that reads entire RNA molecules from end to end in one continuous sequence [71]. Long-read sequencing platforms, such as Pacific Biosciences (PacBio) and Oxford Nanopore Technologies (ONT), enable end-to-end sequencing of full-length mRNA molecules, providing a transformative technology for transcriptome profiling. PacBio sequencing uses an optical system to detect individual fluorescently labeled nucleotides as they are incorporated into a replicating strand [72]. ONT uses an electrical system to measure changes in the electric current as the nucleotide sequence in a single molecule, travels through the pore [73]. Key differences between short and long-read RNA-seq are read length, isoform resolution, accuracy, and cost. Short-read RNA-seq offers high fidelity, cost efficiency, and scalability, making it well-suited for applications such as genome assembly, gene expression analysis, and single nucleotide polymorphism (SNP) detection. However, short-read RNA-seq often struggles to accurately map long repetitive regions or overlapping areas. In contrast, the ability of long-read RNA-seq to read longer sequences overcomes the limits of complex genomic structures and elusive structural variations, delivering deeper genomic insights. Nonetheless, long-read RNA-seq generally incurs higher expenses, exhibits lower raw read accuracy, and demands a more complex data analysis workflow [71].

Although the application of long-read RNA-seq in ALL is still emerging, early studies demonstrate its potential to improve molecular characterization beyond the capabilities of conventional short-read sequencing. By generating full-length transcript reads, long-read technologies enable direct identification of complete fusion transcripts, precise breakpoint characterization, transcript isoform diversity, and alternative splicing events without the need for transcript assembly. These advantages are particularly relevant in ALL, where recurrent gene fusions and aberrant transcript structures play a central role in disease classification and risk stratification. A recent study demonstrated that long-read whole-transcriptome sequencing, together with targeted long-read gene panel profiling, enabled sensitive detection of clinically relevant fusion oncogenes in pediatric B-ALL while simultaneously providing comprehensive transcript-level information. Such approaches may improve the characterization of complex or cryptic rearrangements that are difficult to fully resolve using short-read sequencing alone. However, despite these promising results, long-read RNA-seq currently remains primarily a research tool, and further optimization, large-scale clinical validation, and standardized analytical workflows will be required before its routine implementation in ALL diagnostics [71,74].

## 9. Future Directions and Emerging Trends

### 9.1. Multi-Omics Integration

Multi-omics approaches in ALL integrate genomics, transcriptomics, proteomics, epigenomics, and single-cell data to elucidate the disease’s complexity and molecular heterogeneity. This comprehensive approach has proven valuable in identifying key molecular markers and oncogenic drivers, while also revealing essential biological features and improving leukemia subtype classification. Consequently, these multi-omics strategies have driven the development of more precise, individualized diagnostic and treatment pathways. Advancing leukemia research will require developing computational tools that integrate data from genomics, transcriptomics, proteomics, and the microenvironment to capture the full spectrum of leukemia heterogeneity. Despite the transformative potential of multi-omics, significant barriers persist, particularly in integrating heterogeneous data. Subsequent efforts should address this by developing standardized protocols for seamless data exchange and integration [75].

In a recent study, researchers applied Multi-Omics Factor Analysis to integrate large-scale data of 1231 patients diagnosed with B-cell precursor ALL (BCP-ALL) to overcome the limitations of single-layer analyses. They mathematically linked distinct biological layers (DNA methylation, transcriptional, and drug response data) to reveal hidden, shared patterns of the disease. Their analysis revealed biological patterns related to leukemia subtype, cell growth, and treatment response. Additionally, this multi-omics integration study elucidated how individual and combined data types can inform key biological processes and clinical outcomes, thereby improving risk estimation and disease stratification in pediatric BCP-ALL [76].

The application of multi-omics strategies has revolutionized the understanding of the pathogenetic mechanisms underlying the disease. Nevertheless, the standard of care in ALL continues to rely on a static precision medicine model, in which molecular characterization is largely confined to diagnosis. Adaptive therapeutic decisions that aim at relapse prevention require the full integration of multi-omics approaches through harmonized analytical workflows, the development of clinically oriented multi-omics panels, and clinical validation of integrated longitudinal molecular monitoring in ALL. Multi-omics approaches can reshape the clinical management of ALL, supporting a transition from static risk stratification to dynamic precision medicine strategies [77].

### 9.2. Single-Cell and Spatial Integration

Because ALL is a highly heterogeneous malignancy, integrating scRNA-seq with SRT can provide unparalleled, high-resolution insights into the disease. ScRNA-seq can identify rare, drug-resistant clones and captures the transcriptomic and genetic diversity of malignant cells in unprecedented detail, but it “destroys” tissue architecture during dissociation [40]. On the other hand, SRT technologies track where these specific clones localize in the bone marrow, revealing whether certain resistant populations thrive in specific microenvironmental niches [62,67]. Correlating scRNA-seq data with SRT enables the precise localization and functional characterization of cancer cells’ diversity and their exact bone marrow niches, allowing for highly targeted and individualized treatment approaches to reduce the risk of relapse.

### 9.3. Clinical Translation

It seems that bulk RNA-seq and targeted RNA-seq are likely to remain among the most clinically applicable transcriptomic approaches in the near future. Bulk RNA-seq is increasingly being incorporated into specialized clinical laboratories for comprehensive molecular characterization [3]. While bulk RNA-seq is primarily used for the discovery of novel alterations, targeted RNA-seq panels have emerged as a rapid and increasingly adopted clinical approach. The use of targeted RNA-seq simplifies gene fusion screening and can facilitate the identification of patient-specific molecular markers that may subsequently be incorporated into individualized MRD assays [34].

ScRNA-seq is currently used in specialized research settings to unravel intra-leukemia heterogeneity and identify rare cell populations. However, several challenges such as dropout events, data sparsity, and variability in gene expression persist. scRNA-seq has demonstrated considerable potential for improving disease characterization; its implementation in routine clinical practice will require further reductions in cost, standardized workflows, prospective clinical validation, and regulatory approval [69]. SRT focuses primarily on translational research and currently remains a research tool, with additional technical validation and standardized tissue processing protocols required before broader clinical implementation can be considered [5]. Overall, the degree of clinical readiness differs substantially among transcriptomic technologies. While targeted RNA-seq is already being integrated into routine molecular diagnostics in many specialized laboratories, bulk RNA-seq is increasingly used for comprehensive genomic characterization. In contrast, scRNA-seq and spatial transcriptomics currently remain primarily research technologies whose future clinical implementation will depend on further technical optimization, standardization, analytical validation, and incorporation into evidence-based clinical guidelines.

### 9.4. Complementary Genomic Technologies

Despite the fact that RNA sequencing provides comprehensive transcriptomic information, structural variants that do not generate expressed fusion transcripts may remain undetected. Optical Genome Mapping (OGM) has emerged as a complementary technology capable of detecting balanced and unbalanced structural rearrangements, enhancer hijacking events, and complex chromosomal abnormalities independently of gene expression. Consequently, integrated genomic and transcriptomic approaches may further improve molecular characterization of ALL [78].

### 9.5. Regulatory Approval & Bioinformatics Standardization

Although RNA-seq technologies have demonstrated their clinical value, their widespread implementation remains limited by a lack of standardization. The use of an identical sequencing assay is insufficient without the implementation of validated and reproducible bioinformatics pipelines. These pipelines incorporate clearly defined steps, such as quality control, read alignment, fusion detection, expression quantification, variant annotation, and reporting, to ensure consistent results across different laboratories. Furthermore, each NGS assay must be validated for accuracy, repeatability, reproducibility, limit of detection, and quality metrics prior to clinical use [37]. In addition to analytical validation, standardized clinical reporting is essential to ensure consistent interpretation and communication of molecular findings across laboratories. Consensus recommendations from several organizations, including the Association for Molecular Pathology (AMP), American Society of Clinical Oncology (ASCO), and College of American Pathologists (CAP), provide a framework for the interpretation and reporting of clinically significant genomic alterations, supporting harmonized molecular diagnostics and facilitating the integration of sequencing technologies into routine clinical practice. Future clinical implementation will require validated and standardized bioinformatic workflows that comply with regulatory and laboratory accreditation requirements, together with harmonized quality assurance and reporting standards [79].

Leukemia research cannot advance without adopting breakthrough technologies, such as single-cell and spatial omics, to unravel the complex molecular and cellular mechanisms that drive disease progression and drug resistance. The goal of multi-omics integration is to move from exploratory biomarker research toward precision oncology guidelines. Clinical translation of ALL is shifting from broad, risk-stratified chemotherapy to personalized, multi-omics-driven patient profiling [75,77].

## 10. Conclusions

ALL is a heterogeneous disease characterized by a wide array of genetic lesions, including translocations, deletions, and abnormal chromosome numbers. The evolution of transcriptomics methodologies has transformed leukemia biology understanding from a broad, morphology-based classification into a highly precise, dynamic, and personalized molecular science. Bulk and targeted RNA-seq have vastly improved the molecular subtyping of leukemias; scRNA-seq offers unprecedented resolution of leukemic heterogeneity; and SRT allows researchers to map exactly where malignant, immune, and stromal cells reside within the bone marrow niche.

Summarizing, these transcriptomic methodologies drive a deeper understanding of leukemic pathobiology, clonal heterogeneity, the tumor microenvironment, and treatment outcomes. Additionally, the integration of multi-omics approaches has revolutionized the management of ALL by shifting treatment from broad chemotherapy to precision medicine, enabling highly specific risk stratification, targeted therapies for unique molecular subtypes, and highly sensitive MRD monitoring. The future of transcriptomic profiling in ALL lies not in replacing existing methodologies, but in strategically integrating them to achieve a comprehensive and clinically actionable understanding of leukemic biology.

## Figures and Tables

**Table 1:** Representative studies employing bulk RNA sequencing in ALL.

Reference, First Author (Year)	Study Design	Age Group	ALL Subtype	RNA-Seq Approach	Key Findings
Brown (2020) [3]	Multicenter diagnostic validation study	Pediatric	B-ALL	Whole-transcriptome RNA-seq	Identification of gene fusions, expression signatures and subtype-defining lesions
Tran (2022) [10]	Prospective clinical implementation study	Pediatric	Multiple ALL subtypes	Whole-transcriptome RNA-seq	Detection of cryptic rearrangements and clinically relevant alterations not identified by conventional testing
Dai (2022) [4]	International transcriptomic classification study	Pediatric & Adult	T-ALL	Whole-transcriptome RNA-seq	Definition of transcriptomic T-ALL subgroups and developmental states
Kim (2023) [20]	Molecular classification study	Pediatric & Adult	BCR::ABL1 ALL	Bulk RNA-seq	Identification of distinct transcriptomic classes within BCR::ABL1 ALL
Paietta (2021) [19]	Risk stratification study	Adult	BCR::ABL1-negative B-ALL	Transcriptome profiling	Identification of molecular subgroups associated with prognosis

ALL: acute lymphoblastic leukemia, B-ALL: B-cell acute lymphoblastic leukemia, Bulk RNA-seq: bulk ribonucleic acid sequencing, RNA-seq: ribonucleic acid sequencing, T-ALL: T-cell acute lymphoblastic leukemia.

**Table 3:** Representative applications of RNA sequencing methodologies in ALL.

Method	Key Applications	Representative Findings
Bulk RNA-seq	Molecular classification	Discovery of transcriptomic subtypes
Targeted RNA-seq	Fusion detection	BCR::ABL1-like, DUX4, CRLF2 alterations
scRNA-seq	Clonal heterogeneity	Resistant subclones, leukemic hierarchies
Spatial transcriptomics	Microenvironment profiling	Stromal and immune niche characterization

ALL: acute lymphoblastic leukemia, Bulk RNA-seq: bulk ribonucleic acid sequencing, Targeted RNA-seq: targeted ribonucleic acid sequencing, ScRNA-seq: single-cell ribonucleic acid sequencing.

**Table 4:** Comparative overview of RNA sequencing methodologies in ALL.

Feature	Bulk RNA-Seq	Targeted RNA-Seq	Scrna-Seq	Spatial Transcriptomics
Transcriptome coverage	High	Limited to targets	High	Variable
Resolution	Population-level	Population-level	Single-cell	Spatial/cellular
Fusion detection	Moderate	Excellent	Limited	Limited
Cellular heterogeneity	Poor	Poor	Excellent	Excellent
Spatial information	No	No	No	Yes
Clinical maturity RNA input Seq depth Turnaround time	High 100 ng–1 µg 30–100 M reads/sample 7–14 days	High 10–100 ng 2–10 M reads/sample 3–7 days	Emerging 1000–10,000 cells 20–100 k reads/cell 2–4 weeks	Experimental Tissue section 50–200 M reads/sample 3–6 weeks
Cost	$300–800	$150–500	$1000–3000	$2000–5000
Computational burden	Moderate	Moderate	High	Very high
Main application in ALL	Classification	Molecular diagnostics	Clonal architecture	Microenvironment analysis

The characteristics presented refer primarily to short-read sequencing platforms (Illumina), which currently represent the standard approach for bulk RNA-seq, targeted RNA-seq, scRNA-seq, and most spatial transcriptomic workflows. Values represent typical sequencing depths reported for standard research applications and may vary depending on library preparation protocol, sequencing platform, transcriptome complexity, and study objectives. Approximate turnaround times represent end-to-end research or clinical laboratory workflows and may vary depending on sample batching, sequencing platform, laboratory capacity, and bioinformatic analysis. Approximate research costs per sample, including library preparation and sequencing but excluding personnel, instrumentation, and infrastructure costs. Actual costs vary depending on sequencing platform, assay design, sequencing depth, sample batching, and institutional pricing. ALL: acute lymphoblastic leukemia, Bulk RNA-seq: bulk ribonucleic acid sequencing, Targeted RNA-seq: targeted ribonucleic acid sequencing, ScRNA-seq: single-cell ribonucleic acid sequencing.

## Data Availability

No new data were created or analyzed in this study. Data sharing is not applicable to this article.

## Abbreviations

The following abbreviations are used in this manuscript:

ALL	acute lymphoblastic leukemia
AMP	anchored multiplex PCR
AMP	Association for Molecular Pathology
ASCO	American Society of Clinical Oncology
B-ALL	B-cell acute lymphoblastic leukemia
BCP-ALL	B-cell precursor acute lymphoblastic leukemia
BCR::ABL1	breakpoint cluster region::ABL proto-oncogene 1
bulk RNA-seq	bulk RNA sequencing
CAP	College of American Pathologists
cDNA	complementary deoxyribonucleic acid
DNA	deoxyribonucleic acid
EDTA	ethylenediaminetetraacetic acid
ETP-ALL	early T-cell precursor acute lymphoblastic leukemia
FASTQ	FASTA with quality scores
MPAL	mixed-phenotype acute leukemia
MRD	minimal residual disease
mRNA	messenger ribonucleic acid
NGS	next-generation sequencing
OGM	Optical Genome Mapping
ONT	Oxford Nanopore Technologies
PacBio	Pacific Biosciences
PCR	polymerase chain reaction
Ph-like ALL	Philadelphia chromosome-like acute lymphoblastic leukemia
RNA	ribonucleic acid
RNA-seq	RNA sequencing
scRNA-seq	single-cell RNA sequencing
SNP	single nucleotide polymorphism
SRT	spatially resolved transcriptomics
T-ALL	T-cell acute lymphoblastic leukemia
TCR	T-cell receptor
targeted RNA-seq	targeted RNA sequencing
TKI	tyrosine kinase inhibitor
